# The Obstetrician's Vade Mecum; or, Aphorisms on Natural and Difficult Parturition; the Application and Use of Instruments in Preternatural Labours, or Labours Complicated with Hemorrhage, Convulsions, &c.

**Published:** 1836-10

**Authors:** 


Art. XII.—*The Obstetrician's Vade Mecum; or, Aphorisms on Natural and Difficult Parturition; the application and use of Instruments in Preternatural Labours; or Labours complicated with Hemorrhage, Convulsions, &c.* By Thomas Denman, m.d. &c. *Considerably augmented, and arranged according to the present State of Obstetricy, by* Michael Ryan, m.d. &c. *Ninth Edition. Illustrated with seventeen Plates, and a Portrait of the Author.—London,* 1836. 12mo. pp. 235.

That some important improvements have been made both in the science and practice of midwifery since the time that Denman wrote, is very certain; but it will, we believe, be admitted by all who are conversant with his works, that, even estimating them by the knowledge we now possess, very few practical errors will be detected in them. Various additions are required, and some few corrections, to bring the works of this respected author up to the level of our day; and Dr. Ryan undertakes this task for the well-known "Aphorisms," one of Denman's shortest but not least celebrated publications, as is proved by the fact of its having gone through no less than eight editions. For the purpose of rendering his edition more useful to the student, Dr. Ryan has prefixed the anatomy of the pelvis, and the mechanism of natural parturition. He has also given a minute description of the duties of the obstetrician, and of the assistance to be afforded during natural and difficult parturition. The use of the ergot of rye is also touched upon. Notes are added to the original text where they were required, and a description is introduced of the mode of performing the chief obstetric operations. Dr. Ryan admits that this little work is not intended to supersede systematic treatises on the same subject. Practical conclusions are stated, but the reasonings and facts upon which they are founded are not entered upon.

We shall confine our notice of this edition to the most important alterations and additions that have been made by Dr. Ryan.

The passage of the head through the pelvis, the first position, is accurately described as far as the description goes; but, considering the importance of the subject, we would have had it somewhat more in detail. The second position is dismissed with still more brevity: it is merely stated "that the relative position of the infant and pelvis are the same as the first, but at the other side of the pelvis." Now, as there is much difference of opinion as to the comparative difficulty of the labour, in consequence of the vertex presenting to the right instead of the left acetabulum, we think that Dr. Ryan should have stated his own views upon the subject. Our own experience teaches us nearly to agree with Velpeau* and Osiander,† and almost entirely to differ from Burns.‡ We believe that the interference recommended by the latter, merely on account of the second position of the head, is rarely if ever justifiable. As regards the account given of the manner in which the head passes through the pelvis in the third position,—the occiput to the right sacro-iliac symphysis, the forehead to the left acetabulum,—we must observe that Professor Naegele's§ opinions agree more nearly with our experience than Dr. Ryan's. We do not assert that, in this original position, the occiput never turns into the hollow of the sacrum; but we believe, with Naegele, that much more frequently, at least, the occiput, when it enters the lower part of the pelvic cavity, turns gradually forward, so that the long diameter of the head becomes in relation to the left oblique diameter of the pelvic cavity. And, if the occiput does turn into the hollow of the sacrum, and "the face glide on the anterior and left lateral and inclined plane," the face does *not* "come *under* the neck of the pubes." The forehead rests against the interior of the symphysis pubis, and rises gradually as the occiput is made to descend over the perineum by the uterine power; and it is only when the occiput passes out that the face escapes under the arch. And this indeed Dr. Ryan correctly tells us; but the error consists in his stating that the face "comes *under* the arch of the pubis, *when* the occiput places itself in the curvature of the sacrum.

The "*duties of the obstetrician*" in the lying-in room are, upon the whole, properly stated; but we can hardly believe Dr. Ryan is serious when he says that the practitioner "desires the nurse to pin a napkin round each arm" before he examines per vaginam. All unnecessary parade in the lying-in chamber should be avoided, and this preparation of covering the arms with napkins is no less unnecessary than unusual. In a book, too, intended for the use of students or very young practitioners, we object to the recommendation, "when the pains are slight and teasing, without making any expulsory effort," of giving "a full dose of morphia or opium." We would willingly trust to Dr. Ryan's judgment upon this point, but we fear that a less experienced accoucheur, in acting upon this direction, might fall into the not uncommon error of putting an entire stop to uterine action, by giving too large a dose of opium. "The ergot of rye," Dr. R. says, will "always" effect the removal of a retained placenta. This is surely attributing more certain powers to the ergot than it really possesses. It may effect the object, but it is more likely to fail; and we doubt, at least, whether, in

* Traité des Accouchemens; seconde Ed. t. i. p. 499.
† Handbuch der Entbindungkunst. 1833. Band 3. S. 269.
‡ Midwifery; eighth Edition, p. 393.
§ Mechanismus der Geburt. Heidelberg, 1822.

cases of retained placenta, it would be wise to trust to the ergot at all. Dr. R., too, thinks that the ergot of rye "will supersede the forceps in many instances." We, on the contrary, believe, with Dr. D. Davis, that it is extremely doubtful whether this medicine will *ever* supersede the assistance of the forceps.*

In a note to Denman's Aphorisms on Hemorrhage from attachment of the placenta over the os uteri, Dr. Ryan states that "the contractions of the uterus may be excited by a proper use of ergot, which may be given before or after the hand is introduced into the womb, according to the strength and state of the patient. When there is great debility, the ergot must be given in large or double doses, seldom exceeding half an ounce." Now, granting that the ergot does possess the property assigned to it of producing sudden and violent contractions of the uterus, it could be neither prudent nor safe to give it *before* turning was effected in these cases of hemorrhage; for thus the operation would be rendered more difficult for the practitioner, and more dangerous for the patient. With respect to the "half-ounce" doses which we are "seldom to exceed," we have merely to say, that we never heard the medicine was exhibited in such quantities.

Dr. R. informs us, "it is always to be remembered that the use of the forceps is to diminish the bulk of the head." Now, admitting that where there exists a *moderate* disparity of size between the head of the child and the pelvis of the mother, that the head may be safely and sufficiently diminished in bulk to permit its extraction, "it is always to be remembered," (to use the editor's words, but for another purpose,) that the forceps are at least as frequently applied, on account of inefficient uterine action, &c., where there is no want of proper relation between the size of the body to be passed and the diameters of the cavity through which it is to pass, and where, consequently, no greater pressure upon the head can be necessary or justifiable than secures its being firmly grasped within the blades of the instrument.

To several of Denman's Aphorisms on the use of the Forceps, Dr. Ryan has added judicious notes. He appears to prefer the curved forceps in all cases. The majority of teachers in London recommend the straight forceps, especially for beginners; and we believe this is generally the best form of the instrument.

We do not understand the following statement: "Cephalic version is required when the occiput is turned to the left side, or the reverse. The object is to raise the head into the iliac fossa; press on the abdomen with the other hand, incline the uterus to the side, *and bring down the feet.*" This is certainly *not* "cephalic version;" by which term, the precision of which we are not prepared to defend, is implied a manual operation by which the presenting part is pressed back again, so that the head may descend; or the head itself is to be grasped, and placed in a favorable position. We may observe, that this cephalic version is very rarely practicable. Osiander claims the merit of reviving it from Celsus, and thinks more favorably of the practice than most other modern writers.

We are glad to find Dr. Ryan opposed to a practice which has been

* Madame La Chapelle (Accouchemens, t. i. p. 52,) says of the ergot, "son innocuité est sa plus grande vertu."—Rev.

recently most improperly defended by certain writers in this country. To Denman's Aphorism, "there never can be occasion to separate the arm which presents from the body of the child, and, when this has been done, instead of facilitating, it has impeded the operation" of turning, he adds the following note:

"The arm ought never to be removed, unless when congested so as to fill the os externum and prevent the introduction of the hand; and this rarely happens unless after the infant has been dead for hours or days. The amputation of the arm during the life of the infant is never performed, except by those who are grossly ignorant of medical knowledge; and it is never so swollen during life as to require removal."

To the observation of Dr. Ryan, that the accident referred to rarely happens, we should be inclined to add, that it never takes place.

We cannot perceive anything at all "objectionable" in Denman's cases or doctrine respecting the so-called "spontaneous evolution." Dr. Ryan, in common with most of those who refer to Denman's opinions upon this subject, mistakes their precise meaning.

Upon the subject of "Labours rendered tedious by obliquities of the uterus," Dr. Ryan states, very properly, that the position of the patient should be appropriate to the particular kind of obliquity; but we decidedly object "to breaking down the os uteri with the finger" in these cases. Nature will gradually effect the desired alteration in the situation of the uterus. We state this as a general rule, and admit there may be exceptions to it.

The plates at the end of the volume are very neatly executed, and the subjects illustrated are well chosen for the instruction of the student. In many parts of the work we detect errors, which have apparently crept in from haste or inattention; but we willingly admit that some of the additions to, and emendations of, the Aphorisms of Denman will be useful to the learner.

